# Preaching in Paris

**Published:** 1873-09

**Authors:** 


### PREACHING IN PARIS.

ON a July Sunday morning I found the American Chapel open at half past eleven; the small building was crowded with four hundred hearers, presenting the appearance of a rich congregation of New York. A stranger occupied the pulpit, and with the words, "Touch me not," seemed to engage the attention of the people. Everything was American except that one element, which would have made them all one, the singing; there was not one familiar tune sung, and very few joined in that most interesting and important part of public worship—for song is the mouthpiece of devotion; it is at once preaching, and prayer, and instruction—and more, it is the expression of religious feeling and sentiment. The music was well performed—it was beautiful—but there was no warmth in it. The assembly was not welded together by it, hence left the place without having felt they had been near the gates of heaven. When Captain Hall, near the icebergs in the far north, was about taking his departure on the unknown paths, a parting religious service was held in the little cabin of the Polaris, made up of natives, his own boat's crew, and of others who had come that far, and were about to return to their own homes in America. A gentleman, in describing the interesting meeting, said that the sweetest part of it was in the singing one of the tunes of "Old Times," which was familiar alike to every nationality represented—Americans, Scotch, English and native converts; it seemed to bind them together as one band, and made them all feel as if they were at home in reality; it was, for the time being, a little heaven below. If Dr. Hitchcock will use this suggestion practically, he will find it greatly to his own advantage and that of the little flock far, far from home.

On the first page of the hymn books is pasted a programme of the proceedings—the order of services in English—nothing in French, as if no Frenchman was ever expected to enter the house or take any interest whatever in the proceedings, and that, too, when there are a large number of French Christians in Paris, both of men and women, any one of whom, coming in, would regard this little attention as a recognition of the fact that some interest was felt in them.

At four o'clock in the afternoon the French Protestant Church was open; not of large dimensions—holding some five hundred persons. Very few men were present. All seemed to be from the middle walks of life, and were serious and devout. Gentlemen, before taking their seats, would hold their hats before their faces, as if offering a short prayer, as an introduction into the presence of the great Supreme. The ladies bowed their heads and closed their eyes, and then took their seats, in contrast with the custom at home of taking their seats first.

The services were all in French, continuing about an hour—consisting of singing, prayer, Bible reading and preaching. The clergyman himself reminds one instantly, in size and general features, of the Rev. Dr. John Hall, of New York city; at the same time the head and face bear a strong resemblance to Napoleon the First. He began his sermon leaning on the pulpit cushion with his arms, the hands clasped, speaking in a colloquial tone; as he proceeds he straightens up, the arms are raised, then the hands are separated, the arms move right and left, and the whole man speaks, and with a power, and an earnestness, and a depth of feeling which sway the multitude whithersoever he wills. In the winter time, when the city is full, it is impossible to obtain a seat, unless secured a long time before the services commence. Many American young gentlemen are observed to occupy the same seats for months together; suddenly they will be missed; but after awhile they are seen in their accustomed places, showing that when called from the city for a season they repair to their favorite preachers as soon as they return. Large numbers stand during the entire service, and many, who cannot get in hearing distance, go reluctantly away. The secret of the

Rev. Mr. Bersier's success is much like that of Dr. Hall; you see at once that the whole man is sincere; there is a powerful magnetism about him; you are taken with him before he utters a word, and when he speaks there is a winningness in his utterances which altogether makes it impossible not to be deeply interested. No one thinks about greatness when Dr. Hall speaks; it is the goodness, and the sincerity, and the plainness which carry away the hearts of the hearers; and, when the sermon is over, and you are going home, and can't help thinking about it, there is no thought that you have heard a great sermon, but there is a pleasurable feeling of satisfaction, which makes you go back and love the man who gave rise to it. The people are erecting a large church for him, and they are sorry that, even for a season, he goes to America shortly, and most likely will be present at the Evangelical Association in New York, in October, when some of our readers will, no doubt, have an opportunity of hearing the rising man, for he is yet young—not forty, certainly. There was a very narrow and a very short strip of red observable in the left lapel of the reverend gentleman's coat, so diminutive that one could scarce say whether it was there by accident or design; but seeing it next day on the coat of our landlord, the proprietor of the Hotel Walther, on Taglioni street, very near the Garden of the Tuileries, there seemed to be a significance in it. It is the badge of the Legion of Honor; allowed to be worn as an indication that the wearer had performed meritorious personal service during the reign of the Commune. The captain, in his entire personal appearance, reminds one of Kit Carson, the great Indian fighter of the West. It is well known that Carson, with his small frame and light hair, and soft blue eyes, and gentle, unpresuming manner, looked anything else than a fearless warrior against savages; and so, in the quiet and courteous bearing of Captain

Walther, one would not gather the fact that he dared to stand with his bared bosom, as unflinching as a rock of adamant, against the pointed weapons of an enraged populace; but he did. "The papers," says *Galignani*, "are loud in their praises of the courageous conduct of Captain Walther, of the third battalion of the National Guard, who, during the reign of the Commune, did everything in his power to support the party of order. He was the bearer of the flag when the men of the Hotel de Ville shot down the unarmed people in the Rue de la Paix. The captain, who escaped by a miracle, although he knew himself tracked by the Communists, far from attempting to leave the city, organized a resistance to their tyranny, and saved a million of money from the burning Ministry of Finance, and which was taken to his own hotel in the Rue Castiglioni. He was naturally a mark for the enmity of the Federates, was arrested and conducted to the Prefecture of Police, whence he was happily able to escape."

Captain Walther's father had a decoration of the Legion of Honor from the Emperor Napoleon, and the captain himself treasures one with the greatest care, presented him by the Republic; in addition, the captain possesses a silver medal, presented by Queen Victoria for valuable and heroic services in the Crimean war. At present he is the proprietor of one of the best conducted family hotels in Paris. In many thousand miles of travel we have not met with one more scrupulously clean, with a table which must satisfy the most fastidious American. An experienced traveller, if placed at one fallen from the sky, could not tell that he was not sitting down to the Fifth Avenue or the Brevoort; the seasoning, the preparation is all American.

